# Review of dengue, zika and chikungunya infections in nervous system in endemic areas

**DOI:** 10.1055/s-0043-1777104

**Published:** 2023-12-29

**Authors:** Marzia Puccioni-Sohler, Cristiane Nascimento Soares, Paulo Pereira Christo, Sérgio Monteiro de Almeida

**Affiliations:** 1 Universidade Federal do Estado do Rio de Janeiro, Escola de Medicina e Cirurgia, Departamento de Medicina Geral, Rio de Janeiro RJ, Brazil.; 2 Universidade Federal do Rio de Janeiro, Faculdade de Medicina, Programa de Pós-Graduação em Doenças Infecciosas e Parasitárias, Rio de Janeiro RJ, Brazil.; 3 Hospital Federal dos Servidores do Estado, Rio de Janeiro RJ, Brazil.; 4 Santa Casa BH, Faculdade de Saúde, Programa de Pós-Graduação Stricto Sensu em Medicina-Biomedicina, Belo Horizonte MG, Brazil.; 5 Universidade Federal de Minas Gerais, Faculdade de Medicina, Departamento de Neurologia, Belo Horizonte MG, Brazil.; 6 Universidade Federal do Paraná, Faculdade de Medicina, Departamento de Patologia Médica, Curitiba PR, Brazil.

**Keywords:** Arboviruses, Dengue, Zika Virus, Chikungunya Virus, Neurologic Manifestations, Encephalitis Viruses, Cerebrospinal Fluid, Arbovírus, Dengue, Zika Vírus, Vírus Chikungunya, Manifestações Neurológicas, Vírus da Encefalite, Líquido Cefalorraquidiano

## Abstract

Dengue, zika, and chikungunya are arboviruses of great epidemiological relevance worldwide. The emergence and re-emergence of viral infections transmitted by mosquitoes constitute a serious human public health problem. The neurological manifestations caused by these viruses have a high potential for death or sequelae. The complications that occur in the nervous system associated with arboviruses can be a challenge for diagnosis and treatment. In endemic areas, suspected cases should include acute encephalitis, myelitis, encephalomyelitis, polyradiculoneuritis, and/or other syndromes of the central or peripheral nervous system, in the absence of a known explanation. The confirmation diagnosis is based on viral (isolation or RT-PCR) or antigens detection in tissues, blood, cerebrospinal fluid, or other body fluids, increase in IgG antibody titers between paired serum samples, specific IgM antibody in cerebrospinal fluid and serological conversion to IgM between paired serum samples (non-reactive in the acute phase and reactive in the convalescent). The cerebrospinal fluid examination can demonstrate: 1. etiological agent; 2. inflammatory reaction or protein-cytological dissociation depending on the neurological condition; 3. specific IgM, 4. intrathecal synthesis of specific IgG (dengue and chikungunya); 5. exclusion of other infectious agents. The treatment of neurological complications aims to improve the symptoms, while the vaccine represents the great hope for the control and prevention of neuroinvasive arboviruses. This narrative review summarizes the updated epidemiology, general features, neuropathogenesis, and neurological manifestations associated with dengue, zika, and chikungunya infection.

## Resumo

Dengue, zika e chikungunya são arboviroses de grande relevância epidemiológica em todo o mundo. A emergência e reemergência dessas infecções virais transmitidas por mosquitos constituem um grave problema de saúde pública humana. As manifestações neurológicas causadas por esses vírus têm alto potencial de morte ou sequelas. As complicações que ocorrem no sistema nervoso associadas às arboviroses podem representar um desafio diagnóstico e de tratamento. Em áreas endêmicas, casos suspeitos devem incluir encefalite, mielite, encefalomielite, polirradiculoneurite e/ou outras síndromes do sistema nervoso central ou periférico, na ausência de explicação conhecida. Caso confirmado de arbovirose neuroinvasivo é baseado na detecção viral (isolamento ou RT-PCR) ou de antígenos em tecidos, sangue, líquido cefalorraquidiano ou outros fluidos corporais, aumento dos títulos de anticorpos IgG entre amostras de soro pareadas, anticorpo IgM específico no líquido cefalorraquidiano e conversão sorológica para IgM entre amostras de soro pareadas. O exame do líquido cefalorraquidiano pode demonstrar: 1. agente etiológico; 2. reação inflamatória ou dissociação proteico-citológica, dependendo do quadro neurológico; 3. valor absoluto de IgM específica; 4. síntese intratecal de anticorpos IgG específicos (dengue e chikungunya); 5. exclusão de outros agentes infecciosos. O tratamento das complicações neurológicas visa melhorar os sintomas, enquanto a vacina representa a grande esperança para o controle e a prevenção das arboviroses neuroinvasivas. Esta revisão narrativa resume a atualização da epidemiologia, características gerais, neuropatogênese e manifestações neurológicas associadas à infecção pelos vírus da dengue, zika e chikungunya.

## INTRODUCTION

Arboviruses are viruses transmitted to the vertebrate host by the bite of arthropod vectors, especially mosquitoes and ticks, and can be maintained in wild and/or urban cycles. Among the arboviruses, the main families that cause disease in humans are Flaviviridae, Togaviridae, and Bunyaviridae ( Table 1 ). The phenomenon of urbanization and climate change contribute to the wide distribution of vector mosquitoes. These factors facilitate the co-circulation of the agents across the world and, consequently, the appearance of serious diseases caused by them such as neurological disturbances. 1

**Table 1:** Main families and genera of arboviruses

Family	Genus	Virus / Disease
Flaviridae	Flavivirus	Dengue, Zika, Yellow fever, West Nile, Japanese encephalitis, St. Louis encephalitis, Rocio, tick-borne encephalitis
Togaviridae	Alphavirus	Chikungunya, Mayaro, Ross river, Venezuelan equine encephalitis, eastern equine encephalitis, western equine encephalitis,
Bunyaviridae	Orthobunyavirus Phlebovirus	Oropouche, La Crosse Toscana, Rift valey fever
Reoviridae	Coltivirus	Colorado Rick fever

Arboviruses of great importance in public health include dengue (DENV), zika (ZIKV), and chikungunya (CKIKV) viruses. These are transmitted by female mosquitoes of the genus *Aedes (Ae. aegypti and Ae. albopictus* ) after a blood meal in a viremic human host. Since 2015, there has been a triple epidemic caused by DENV, ZIKV, and CHIKV viruses in Brazil. In 2022, there was an explosion of cases and deaths from DENV: 1,450,270 probable cases of the disease (an increase of 162.5%) and 1,016 deaths (an increase of 313%) compared with 2021. There was also an increase in CHIKV (78.9%) and ZIKV cases (98.8%) in 2022 when compared with the year 2021. 2

The involvement of the nervous system occurs in ∼1–21% of patients with DENV, 16% of patients with CHIKV, and an estimated 13.4 to 33.3% of patients with ZIKV. 1 3 Regarding the neurological manifestations associated with DENV, there is a relevant suspicion of underreporting, since these complications can occur even in patients with few or no other symptoms. Only 25% of DENV cases are symptomatic, compared with 70% of symptomatic people infected with CHIKV. The main neurological manifestations associated with DENV, ZIKV, and CHIKV include encephalitis, myelitis, meningitis, congenital malformation syndrome (ZIKV), encephalopathy, cerebromeningeal hemorrhage (DENV) and autoimmune diseases such as Guillain-Barrè syndrome [GBS], optic neuromyelitis, acute disseminated encephalomyelitis [ADEM], polyneuropathies and mononeuropathies. 1

The diagnosis of neuroinvasive arboviruses (DENV, ZIKV, CHIKV) must include all acute viral infections or post-infectious conditions of the central and/or peripheral nervous system confirmed by the presence of antigens, viral and specific antibodies detection in serum and/or cerebrospinal fluid (CSF). 1 3

In a previous study, Mello et al. (2020) 3 found 31% of neuroinvasive arboviruses in adult patients with suspected viral infection of the central nervous system (CNS) or postinfectious syndromes living in the state of Rio de Janeiro, Brazil. Of the total group, 17% of neurological conditions were associated with CHIKV, and 14% were associated with DENV. The laboratory diagnosis was based on the combined use of molecular techniques (reverse transcription RT-PCR) and immunological (IgM) tests for DENV, CHIKV, and ZIKV in CSF/serum, and evaluation of intrathecal synthesis of anti-DENV and anti-CHIKV IgG antibodies. 3

Considering the epidemiological relevance of the arboviruses with potential nervous system impairment for public health in Brazil and in the world, as well as the challenges in the diagnosis and treatment of complications, our aim is to carry out a narrative review on the DENV, ZIKV and CHIKV and neurological involvement.

## DENGUE

DENV fever is the most common arboviral disease worldwide, been endemic in more than 100 countries. The Philippines, Vietnam, India, Colombia, and Brazil were reported to have the highest number of DENV cases. 4

DENV is a flavivirus belonging to Flaviviridae family. It is an RNA virus enclosing three types of structural proteins including capsid, envelope, and membrane, and seven non-structural (NS) proteins. 5

In 2013, a new DENV serotype was discovered in Malaysia. This DENV5 is prevalent in forests of Southeast Asia, and it is believed that its emergence happened due to the high dengue mutation frequency. 5 Despite this, serotypes 2 and 3 are still more associated with neurological manifestations. 6

### Clinical manifestations

DENV fever can manifest an asymptomatic form or severe hemorrhagic symptoms/hypovolemic shock. This occurs after 4–10 days of the *Aedes* mosquito bite. DENV fever is characterized by a rapid onset of fever, headache, retro-orbital pain with severe myalgia, and arthralgia. For practical reasons, in 2009 WHO categorized DENV fever as non-severe and severe, based on a set of clinical and/ or laboratory parameters. The non-severe group was classified also into those with warning signs and those without warning signs ( Table 2 ). This new classification had made it easier to decide how intense clinicians should observe and treat DENV fever patients. 7

**Table 2:** Dengue classification according warning signs and severity

Probable dengue	Warning signs	Criteria for severe dengue
Live in /travel to dengue endemic area.	• Abdominal pain or tenderness • Persistent vomiting • Clinical fluid accumulation • Mucosal bleed • Lethargy, restlessness • Liver enlargment >2 cm • Laboratory: increase in HCT concurrent with rapid decrease in platelet count	Severe plasma leakage leading to: • Shock (DSS) • Fluid accumulation with respiratory distress
Fever and 2 of the following criteria: • Nausea, vomiting • Rash • Aches and pains • Tourniquet test positive • Leukopenia • Any warning sign		Severe bleeding as evaluated by clinician
		Severe organ involvement • Liver: AST or ALT > = 1000 • CNS: Impaired consciousness • Heart and other organs

### Neurological manifestations

The exact incidence of neurological involvement in DENV infection is still variable, ranging between 0∙5% and 5∙4% in southeast Asia to 21% in Brazil. 8 We will describe DENV neurological manifestations based on their specific mechanism of neuropathogenesis since they seem to be strictly related ( Table 3 ).

**Table 3:** Dengue neuropathogenesis and neurological complications

Metabolic disturbance	Viral invasion	Autoimmune reaction	Autoimmune reaction and viral invasion
Encephalopathy Hypokalaemic paralysis	Encephalitis Meningitis Myelitis	Acute disseminated encephalomyelitis Neuromyelitis optica Post-infectious encephalopathy Guillain Barré syndrome Optic neuritis	Myelitis Myositis

#### *Metabolic disturbance*

Encephalopathy is the most common neurological manifestation caused by DENV. It is characterized by reduced consciousness secondary to complications such as liver failure, renal impairment, electrolyte imbalance, hypoxia, shock, and coagulation disorders. 8 Usually there are no focal abnormalities in electroencephalography (EEG) or in brain neuroimaging, but sometimes diffuse cerebral edema can be found. CSF analysis is normal.

Hypokalemic paralysis due DENV infection is rarely reported. This diagnosis should be suspected when patients present an acute onset of flaccid quadriplegia without any cranial nerve palsy. A serum potassium level of 3 mmol/liter or below confirms the diagnosis. 9 It is a treatable condition, with complete recovery after potassium supplementation.

#### *Direct viral invasion*

In studies using mice infected by DENV, the cytokine response led to a breakdown of the blood-brain barrier, allowing the virus to invade the CNS. 10 In this regard DENV enters in the brain parenchyma via the bloodstream.

DENV is one of the leading causes of viral encephalitis in endemic areas. It usually develops from three to seven days from the start of the fever. It is defined by:

- presence of fever;
- acute signs of cerebral involvement such as altered consciousness or personality and/or seizures and/or focal neurological signs;
- reactive IgM dengue antibody, NS1 antigen, or positive dengue reverse transcription polymerase chain reaction (RT-PCR) on serum and/or CSF;
- exclusion of other causes of viral encephalitis and encephalopathy. 11

Brain image findings are variable. It may be normal, or reveal hemorrhages, diffuse cerebral edema, and focal abnormalities. Hyper-intense lesions at the globus pallidus, hippocampus, thalamus, and internal capsule are described. The double-doughnut sign can also be shown on the MRI brain. These lesions are hyperintense on T2 and FLAIR images, with central hemorrhages, affecting usually bilateral thalamic regions. 12 Therefore, generalized dystonia/parkinsonism may develop, attributable to this bilateral thalamic involvement. Movement disorders have been observed in 11% of DENV encephalitis patients. 13

In contrast to DENV encephalitis, meningitis secondary to this infection is rarely seen in adults. 14

Myelitis is a less common complication, ranging from 9.5% to 15%, and presenting mainly with sensorimotor disturbance of the lower limbs and urinary retention. Myelitis can be determined by DENV infection secondary to virus invasion or by an autoimmune reaction, evolving between seven and 30 days following the infection. The viral neurotropism mechanism could be confirmed by the detection of local synthesis of dengue antibodies in CSF. 15

DENV myositis is also a rare manifestation, being associated with a viremic phase, ranging from mild, proximal, asymmetrical weakness and myalgia of the lower limbs to severe, rapidly progressing weakness, even with respiratory insufficiency. Elevated creatine phosphokinase levels, compatible electromyography, and muscle biopsy can provide the correct diagnosis. 16 The pathogenesis of myositis remains unclear, between virus invasion or an autoimmune reaction. However, some studies have demonstrated the presence of DENV in the striated muscles, reinforcing the first mechanism. 17

#### *Autoimmune diseases*

After 1–3 weeks of the onset of infection, a delayed neurological complication can occur secondary to cell-mediated immunological reactions. Dengue infection may trigger an abnormal immune response, in which antibodies can cross-react with the myelin, via molecular mimicry. GBS, ADEM, neuromyelitis optica, myelitis, or simply a post-infectious encephalopathy are examples of this autoimmune reaction. 18

GBS is the most common manifestation in the peripheral nervous system, secondary to DENV infection (ranging from 20 to 30%). 19 It usually develops its symptoms between four and nineteen days after the infection onset and has similar characteristics and prognosis to that caused by other infections. Soares et al reported that 46.6% of all their GBS cases had dengue-positive IgM, but with little to no clinical symptoms of DENV infection. 20 Therefore, DENV infection should always be investigated in GBS cases. Mononeuropathies as long thoracic neuropathy, optic neuritis, oculomotor palsy, and phrenic neuropathy have been also related. 21 22 23

ADEM generally occurs after remission of the febrile period, with seizures, altered sensorium, and focal neurological deficits. The diagnosis is suggested by magnetic resonance imaging (MRI), which shows white matter lesions on T2-weighted and FLAIR images in the centrum semiovale, corona radiata, corpus callosum, thalamus, and thoracic/cervical segments of the spinal cord. 9

**Figure 1:**
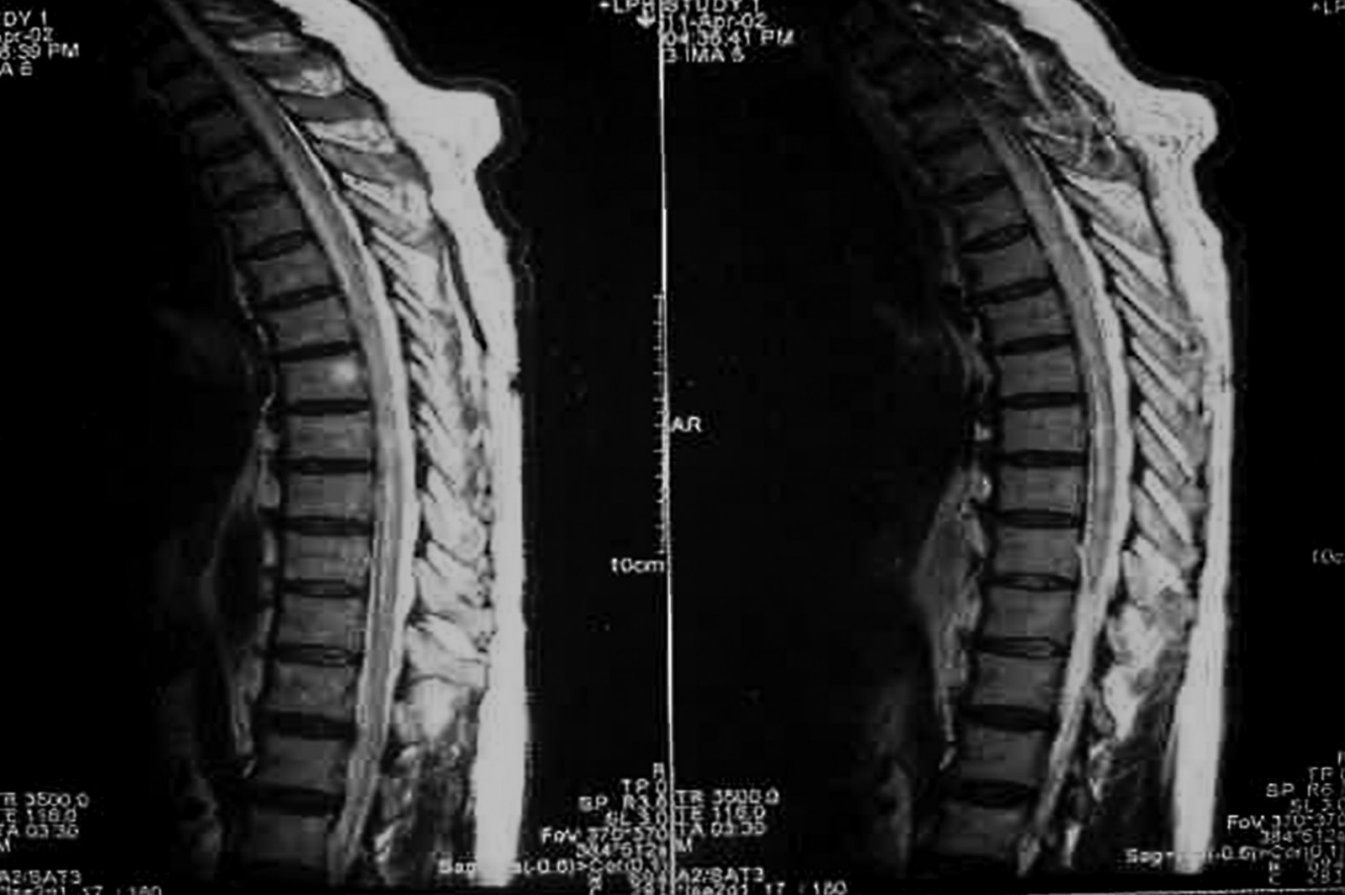
Magnetic resonance image (sagittal plane, T2 sequence): Extensive enhanced signal at dorsal spinal cord in a patient presenting acute myelitis caused by dengue infection.

**Figure 2:**
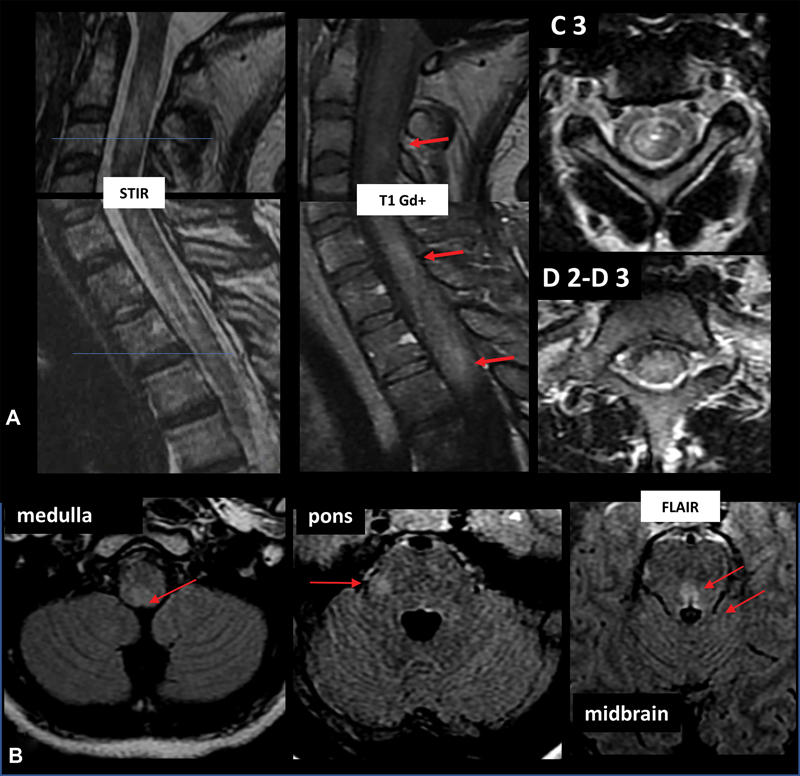
18-year-old woman, with a confirmed diagnosis of dengue virus type 1 (RT-PCR) in blood, presenting with transverse myelitis (cervical and thoracic) and brainstem syndrome compatible with neuromyelitis optica spectrum disorder. CNS lesions depicted on MRI exams: (A) spine and (B) in the brain. (A) Longitudinally extensive involvement of cervical spine (with scattered enhancement), high thoracic spinal cord (over three vertebral bodies extension) MRI. (B) Lesions with high signal on FLAIR were observed on the right dorsal medulla and middle cerebellar peduncle, and in the midbrain (periaqueductal).

DENV-associated longitudinally extensive transverse myelitis (LETM) is uncommon ( Figure 1 ). As LETM is characterized by an intramedullary spinal cord lesion extending over more than three contiguous vertebral body segments on MRI, it is also part of neuromyelitis optica spectrum disorder (NMOSD) ( Figure 2 ). 24 Rare cases of DENV-associated NMOSD, and positive AQP4-IgG are described in the literature. 25 It is still unclear if the association NOMSD- DENV is coincidental, causal, or triggering.

### Diagnosis

Laboratory diagnosis of DENV infection is based on three methods: direct viral detection (RT-PCR or virus isolation), virus products detection (NS1 protein), or immunology (detection of virus-specific IgM and IgG). 26 Except for RT-RT-PCR, which is sensitive, specific, fast, and cheaper, virus isolation is not practical, as it can take days to weeks to perform.

Laboratory tests should be selected according to the onset of illness. Until five days of symptoms, detection of viral RNA by RT-PCR, or detection of viral antigens such as NS1 are the choice methods. After 4–5 days, although NS1 antigens can persist, serological assays should be used. IgM antibodies in serum or the increase of dengue IgG antibody titer in paired samples can be detected. Dengue IgM antibodies usually have a peak of 10–14 days and disappear ∼3 months in serum. The sensitivity of dengue IgM in CSF ranges from 0–73% and the specificity from 97–100%. 6 Although a typical DENV infection CSF pattern presents pleocytosis and hyperproteinorhachia, a normal CSF examination does not exclude the possibility of this infection. In neurological cases, the combined use of NS1 Ag and specific IgM antibodies in CSF can improve the sensitivity of diagnosis to 92%. 27 The major problem of serological tests is their cross-reactivity with other flaviviruses, and often acute and convalescent samples are required and also carrying out the plaque reduction neutralization.

### Treatment

Currently, there is no specific treatment for DENV infection, the therapeutic options are purely supportive. Judicious fluid resuscitation during the critical phase of dengue is fundamental. In neurological cases, besides adequate hydration, monitoring of consciousness, airway protection, anti-seizure medications in case of seizures, and anti-raised intracranial pressure measures should be implanted depending on the clinical picture.

In encephalitis/encephalopathy cases, the use of high-dose steroids has been advocated in isolated case reports. 28 In addition, pulsed steroids may be helpful in ADEM, NMOSD and myelitis patients. Similarly, to other GBS causes, in DENV, intravenous immunoglobulin (IVIg) is also recommended. 8

The development of a DENV vaccine is essential. The first licensed DENV vaccine in Brazil was Dengvaxia. This vaccine had presented two important limitations: a relatively low efficacy (60.3%) and its restriction for use in individuals with prior dengue infection. 29 Recently, QDENGA® or TAK-003 by Takeda Pharmaceuticals has been licensed. It can be applied in four to 60-year-old individuals, regardless of their prior DENV exposure status. It should be administered subcutaneously, in two doses three months apart. 30

## ZIKA

ZIKV was isolated from the blood of a sentinel rhesus monkey located in the Zika Forest of Uganda in 1947. For six decades ZIKV remained confined to Africa and Asia, causing sporadic outbreaks. In 2007, the first major ZIKV outbreak was reported from Yap Island. 31 The virus spread over the Pacific Ocean and caused an outbreak in French Polynesia in 2013–2014 and arrived in the Americas between 2013 and 2015. 32 Brazil was the most affected country, with 440,000 to 1.3 million cases. 33 On February 1, 2016, the World Health Organization declared a Public Health Emergency of International concern. 34 However, since the end of 2016, transmission of ZIKV has declined significantly in the world. Just under 30,000 cases reported in Brazil in 2018 compared with more than 500,000 during the outbreak in early 2016. 35

ZIKV is an arbovirus belonging to the genus Flavivirus of the family Flaviviridae. Two major ZIKV genotypes have been identified and exhibit few different amino acid sequences. The African-ZIKV genotype has caused sporadic or recurrent infections in West African countries and the Asian-ZIKV genotype has circulated in Southeast Asia, the Pacific Islands, and the Americas, causing major outbreaks. 33

Although ZIKV is considered primarily a vector-borne disease, there is evidence of non-mosquito transmission to humans including mother to fetus, nosocomial, by transfusion, through bone marrow or organ transplantation, sexually, breastfeeding, saliva, urine, and monkey bites. 36 ZIKV is the first arbovirus to be detected in the semen. Vertical transmission of ZIKV can occur in all three trimesters of pregnancy regardless of the presence or absence of symptoms in the mother. Approximately 26% of infected mothers transmit ZIKV to their fetuses. The risk of developing birth defects was highest among women who were infected during the first trimester and lowest among women who acquired the infection during the third trimester. 35

### Clinical manifestations

Most people (80%) infected with ZIKV do not develop symptoms, which may occur 3–14 days after infection. These consist of rash, fever, conjunctivitis, muscle and joint pain, malaise, and headache. during 2–7 days.

### Neurological manifestations

ZIKV infection is associated with a wide spectrum of neurological complications as congenital Zika syndrome (CZV). 34 In adolescents and adults, GBS, meningitis, encephalitis, myelitis, radiculitis, ADEM, ophthalmological and vascular complications have been rarely reported. 32 37 38

#### *Congenital zika syndrome*

The association of ZIKV infections with neonatal complications was recognized in late 2015 when the number of neonatal microcephaly cases increased during the ZIKV outbreak in Brazil. 39 The term CZS has been adopted to describe a set of symptoms and signs in children, affecting mainly CNS along with other systems. CZS occurs in 5 to 14% and microcephaly in 4 to 6%. 35

About 3,700 cases were reported in 27 countries in the Americas by 2018. Brazil concentrated 80% of them. 40 According to data from a cohort of Brazilian newborns, the mortality rate of confirmed or probable congenital infection by ZIKV is 4–6%. 41 ZIKV is now considered the newest member of the TORCHS (toxoplasmosis, rubella, cytomegalovirus, herpes simplex, and syphilis) pathogens. 35

CZS has been mainly divided into structural and functional lesions. The structural lesions include fetal brain disruption sequence like severe microcephaly, collapsed skull, premature closure of fontanels and redundant scalp skin (rarely seen in other congenital infections), brain abnormalities like subcortical calcifications, decreased myelination, ventriculomegaly, cerebellar hypoplasia, and neuronal migration disorder. Intrauterine growth restriction, congenital contractures and several ocular structural lesions including macular scarring, glaucoma, optic nerve atrophy, intraocular calcifications, cataracts, chorioretinal atrophy, pigmented retinal mottling may also occur. 35 The functional lesions comprise seizures, visual impairment, and hearing loss. Developmental delay and pyramidal and extrapyramidal lesions such as hypertonia, dysphagia, and movement abnormalities. 35

Complications are reported even in children without any clinical or radiological abnormalities at birth. The motor scores of children with prenatal exposure to ZIKV without microcephaly were significantly lower than those of controls. 42

#### *Guillain-Barré syndrome*

GBS has been the most common neurologic complication associated with ZIKV in adolescents and adults. In French Polynesia, the incidence rate of GBS increased 20-fold during the outbreak of 2013–2014. 32 In the Americas, the incidence of ZIKV disease was associated with changes in the incidence of GBS cases to a level between 2 and 10 times as high as baseline. 43

GBS associated with ZIKV has been found to occur sooner after the infection onset (6–10 days). 44 Nerve conduction showed mixed results: predominantly acute motor axonal neuropathy in a study done in French Polynesia and acute inflammatory demyelination polyradiculopathy in two other studies conducted in Brazil and Colombia. Mechanical ventilation was needed for almost one-third of the affected patients. 32 Prognosis of ZIKV–GBS does not seem to significantly differ from traditional GBS. 44 Two major studies showed close to 60% of patients becoming ambulatory at 3 months. 32 45

Whether GBS occurs as a consequence of direct viral infection (suggested by the short interval between rash and GBS) or results from an autoimmune, cross-reactive mechanism targeting neurons and glial cells (suggested by the positivity of antiganglioside antibodies in ZIKV-associated GBS) remains to be determined. 46

#### *ADEM and transverse myelitis*

Transverse myelitis and ADEM can be seen concomitantly or shortly after the onset of infection (parainfectious) or can occur after a period of latency (postinfectious) resulting from immune-mediated reactions against the virus. Neuroimaging findings in ZIKV-related ADEM do not differ from those caused by other etiologies. 37 47 48

**Figure 3:**
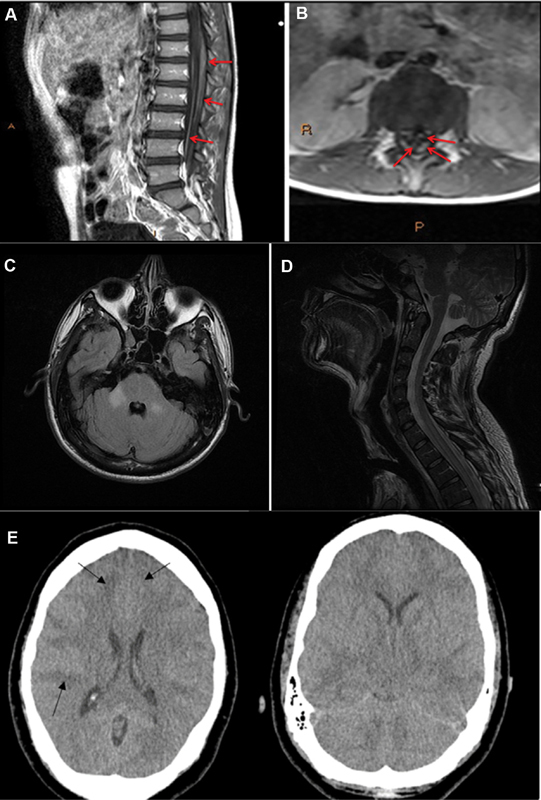
**(** A and B) 7-year-old boy that presented an encephalomyeloradiculitis. (A) Sagittal T1 post-contrast image. Enhancement of the distal cord/conus medullaris and cauda equina. (B) Axial T1 post-contrast image, showing thickening of nerve roots, primarily of the posterior nerve roots. The arrows indicate the thickening of the roots. (C and D). 30-year-old man with zika encephalomyelitis. (C) MRI (axial plane, T2-FLAIR sequence) shows bilateral middle cerebellar peduncle hyperintensity (D) MRI (sagittal plane, T2- FLAIR sequence) shows extensive hyperintensity at dorsal spinal cord. (E) Fatal encephalitis associated with zika virus infection in an adult. Computerized tomography (CT) head showing diffuse cerebral edema with enhacement of basal cisterns and sulcal markings. Observe diffuse subcortical hypodensities, mainly on frontal regions (arrows).

Spinal cord involvement in ZIKV can vary according to the different forms of involvement. 45 Clinical symptoms depend on the location and extension of the lesion. Longitudinally extensive myelitis has been described 49 ( Figure 3 ). A mean latency period of 10.5 days (1–96 days) was found in a review of 19 cases of transverse myelitis, encephalitis, and ADEM. 46 47 48 In one report of transverse myelitis after ZIKV infection, an anti-myelin oligodendrocyte glycoprotein antibody against the myelin sheath was detected, favoring an autoimmune-mediated neurotoxicity. 48

The role of preexisting immunity against other arboviruses (CHIKV and DENV) from prior infection may lead to augmented auto-inflammatory response against the neural tissue after subsequent ZIKV infection. This was hypothesized in a patient who developed ADEM and GBS after ZIKA infection. 45 46

#### *Encephalitis*

Patients may have involvement of the meninges, brain parenchyma, spinal cord, and peripheral nerves in various combinations, as in other flaviviruses infection ( Figure 3 ). A study performed in Colombia identified a total of six children with encephalitis. Lymphocytosis of the CSF was present in all cases, and higher cytokine levels were found in the CSF when compared with plasma levels of one patient, suggesting local inflammation. 50

ZIKV-associated encephalitis symptoms have been reported to appear 1–8 days after infection symptom onset. Clinically, patients usually present with seizures, somnolence, headache, and vomiting due to increased intracranial pressure, motor and sensory dysfunction, cranial nerve palsy, nuchal rigidity, and focal deficits. Symptoms may evolve into deep coma and brain death. Neuroimaging is not specific. 51 52

#### *Cerebrovascular disease*

Rare cases have been described of ischemic and hemorrhagic stroke. 52 Previous studies have demonstrated that endothelial cells of the blood-brain barrier are permeable to ZIKV infection and replication, and they may penetrate the endothelium, causing neuronal tissue damage. Considering that Zika has tropism and may infect endothelial cells, as demonstrated in animal models, and due to its phylogenetic relationship to DENV it is reasonable to admit a correlation between this ZIKV and strokes or brain hemorrhage. 52

### Diagnosis

During the first 5 days of disease onset, the main method of laboratory diagnosis is the detection of RNA by coupled RT-RT-PCR in the blood. However, owing to the short period of viraemia, a negative result does not rule out the possibility of the disease and a urine RT-RT-PCR test is an alternative. ZIKV RNA has been detected in urine up to 20 days after infection.

Serological diagnosis is undertaken by detecting IgM antibodies specific to ZIKV by enzyme immunoassay (EIA) in two serum samples collected at the beginning of symptoms and 14–21 days later to demonstrate seroconversion or a 4-fold increase in the antibody titer. In some cases, a positive result for IgM must be confirmed by a more specific technique such as plaque reduction neutralization (PRNT) which is the gold standard for differentiating anti-flavivirus antibodies as it is relatively specific in primary flavivirus infection. However, PRNT is expensive and can only be undertaken in highly specialized laboratories that require certification to use live viruses. 41 Specific IgG is detectable shortly after appearance of IgM and generally persists lifelong. Among patients living in flaviviruses endemic areas with previous infection with flaviviruses, a subsequent infection with another flavivirus may produce IgG against multiple flaviviruses very rapidly, thus limiting the value of checking IgG in such patients. 53

The viral detection by isolation or RT-PCR in tissues, blood, CSF, urine or other body fluids confirms the diagnosis. The duration and kinetics of detection of ZIKV RNA in CSF are unknown because CSF samples are not routinely collected and analyzed for ZIKV diagnosis. In patients with GBS associated with ZIKV, albuminocytological dissociation from CSF occurs in ∼80% of reported cases. CNS-associated complications of ZIKV, such as encephalitis and myelitis, appear to have a neuroinflammatory profile due to the presence of lymphocytic pleocytosis and hyperproteinorrachia. 50

### Management

Currently, no specific antiviral or vaccine is available against ZIKV infection, and the management of ZIKV cases relies on symptomatic care. 54 Newborns with CZS should be followed up by a multidisciplinary team. Neurological examination and ophthalmological examination must be undertaken should be performed early, when possible, and repeated during follow-up. Brainstem auditory evoked potential should be performed. Early stimulation such as motor physiotherapy and speech therapy is essential. 54 The management of ZIKV-associated GBS was extrapolated from experience in treating classical GBS. Treatments include supportive care and intravenous immunoglobulins or plasmapheresis. Some patients who had ZIKV encephalomyelitis were treated either with high doses of corticosteroids (methylprednisolone) or IVIg, however, there is no evidence to support the use of these drugs in ZIKV-associated encephalitis. 55

## CHIKUNGUNYA

CHIKV was first isolated in 1953. The first documented human outbreaks were in southern Asia during the 1960s to 1970s. During the past 50 years, numerous CHIKV re-emergences have been documented in both Africa and Asia, with irregular intervals of 2–20 years between outbreaks. CHIKV has caused over 70 epidemics between 1952 and 2018. 56 The word “chikungunya” originates from the Makonde language (Tanzania and Mozambique) meaning “that which bends up”; this refers to the contorted posture due to the debilitating arthralgia often occurring in the acute phase of infection. 57

CHIKV is an alphavirus (genus Alphavirus, family Togaviridae), which comprises enveloped, positive single-stranded-RNA viruses, that is primarily transmitted to humans through the bite by infected *Aedes aegypti* or *Aedes albopictus* mosquitoes, and occasionally from mother to child. This broad host tropism of CHIKV indicates that the virus uses a ubiquitously expressed receptor to infect cells. 58 Neurons, astrocytes, and oligodendrocytes (but not microglia) are susceptible to CHIKV infection in vitro. 59 60 CHIKV has also previously shown tropism to the choroid plexus, meninges, and ependymal cells of fetuses. 61 The genetic variability of CHIKV might play a key role in determining the course of neuropathogenesis. Neurological disease secondary to CHIKV has been reported in areas with both ECSA (or ECSA-diverged Indian Ocean lineage) and Asian strains, but whether these strains have differing neurovirulence is unknown. 62 Neurological disease associated with CHIKV is being reported increasingly, especially in large outbreaks with the introduction of the virus to other geographic areas as South American. 62

### Clinical manifestations

CHIKV fever can evolve in three phases: acute with persistent symptoms for up to 14 days, subacute with the sustainment of symptoms for up to three months and chronic, a phase in which symptoms last for more than three months. 63

Classical CHIKV fever symptoms are fever in 98%, arthralgia/ arthritis in 71% and rash in 45%; the complete triad was present in 36% ( Table 4 ). Other symptoms include headache, retro-ocular pain, back pain, nausea, or vomiting. 63

**Table 4:** Neurological complication in Chikungunya virus infection

Neurological complication	Frequency (%)	Time after systemic symptoms (days)	Pathophysiology
Encephalitis and encephalopathy	45	0 to 13	direct Viral infection or immunomediated
Myelitis and myelopathy	Not known (2.4% ?)	0 to 21	direct Viral infection or immunomediated
Peripheral nervous system	9	3 to 17; post infection	direct Viral infection or immunomediated
Meningitis	rare	acute	direct Viral infection
GBS	?	3 to 17; post infection	immunomediated
Cognitive impairment	?	post infection	direct Viral infection or immunomediated
ADEM	?	post infection	immunomediated

Abbreviations: ADEM, acute disseminated encephalomyelitis; GBS, Guillain-Barré syndrome.

### Neurological manifestations

Approximately 0.1% (1 case per 1000 *)* of all CHIKV infections developed neurological disease. 62 However, a previous study conducted in India found 16% of neurological complications in 300 cases of CHIKV infection. 64 Neuro-CHIKV is a severe form of CHIKV infection with a broad spectrum. The mechanisms by which CHIKV affects the nervous system, whether direct injury and/or immune-mediated injury have not been fully. 56 CNS and PNS in association with CHIKV infection can be seen in the same patient. CHIKV infection is associated with complex neurological diseases causing encephalomyelopathy, myeloneuropathy, and encephalomyeloneuropathy, in addition to encephalitis, optic neuropathy, retinitis, and GBS. 62 65

Most of the patients had an unfavorable outcome. 62 Neurological complications are a major cause of intensive care unit admission and death in CHIKV fever patients. 65 Of the CHIKV neurological cases reported, 93.0% were in adults and children infected directly via mosquito and 7.0% were in neonates infected vertically from mother to child. 62

Neurological symptoms were preceded by classical CHIKV fever symptoms; usually, they start on average 10 days after symptom onset (median 7, range 1–53). 65 The neurological complications of CHIKV infection are summarized in Table 4 .

#### *Encephalitis*

Encephalitis is the most reported neurological complication associated with CHIKV. The disease generally starts in the acute phase of CHIK fever. 65 Symptoms begin between 0 and 13 days following the onset of systemic manifestations of infection. 62

#### *Spinal cord disease*

Spinal cord disease typically presents 0 day to 3 weeks after the first general classical symptoms of the infection (fever, arthralgia, or rash). It usually occurs as part of more widespread neurological disease, myelopathy and myeloneuropathy were reported each in 14%. 62 64 An immune-mediated pathogenesis has been proposed, including molecular mimicry, polyclonal T and B cell activation, and immune complex deposition. 66 No deaths have been reported for patients with pure myelopathy syndrome. 62

#### *Cognitive impairment*

A study with volunteers aged from 60 to 90 who had been affected by CHIKV showed that CHIKV infection may cause long-term cognitive decline in aged people and might be a risk factor for future dementia in this population. CHIKV-affected participants had a poorer performance in global cognitive function assessed by MoCA, and by specific cognitive tests for general executive functions, cognitive flexibility, inhibitory control, processing speed, semantic memory, and episodic memory. 67

#### *Acute disseminated encephalomyelitis*

CHIKV, like other acute viral infections, can trigger an ADEM involving the brain parenchyma and spinal cord. It is thought to be an immune-mediated disease, rather than due to direct viral invasion. The diagnosis of this monophasic illness is usually based on finding focal or multifocal, poorly demarcated white matter demyelinating lesions on MRI. 68

#### *Guillain-Barré syndrome*

GBS tends to occur subacutely after a symptom-free interval. 65 The time interval between CHIKV infection and the onset of neurological features describes a prodrome of 3 to 17 days, compatible with a parainfectious or postinfectious syndrome. 62 Electroneuromyography (ENMG) was performed in all GBS-MFS cases and showed a demyelinating (2/6) or combined axonal/demyelinating pattern (4/6). 65

#### *Perinatally acquired neurological disease*

Most of the evidence on CHIKV causing neonatal disease relates to transmission in the intrapartum period, rather than earlier in pregnancy. 62 Maternal CHIKV infection earlier in pregnancy does not appear to affect the fetus. 69 A wide range of severe manifestations has been described as perinatal encephalopathy or brain hemorrhage. 62 There was no protective effect of caesarean. 69 70 The neurological effects of vertically transmitted CHIKV may not be obvious at birth, emphasizing the importance of follow-up of this cohort.

#### *Less frequent neurological manifestations*

Meningitis caused by CHIKV is infrequent, it should be considered in persons who have traveled to endemic areas and have symptoms suggestive of acute meningitis and CSF with findings of acute viral meningitis. Other less frequent neurological manifestations of CHIKV are behavioral changes, sensorineural hearing loss, stroke, cerebellitis, and bilateral total ophthalmoplegia. 62

### Diagnosis

In endemic areas, CHIKV should be tested for in all patients presenting with acute neurological disease and all pregnant women presenting with fever, arthralgia, or rash; neonates with suspected infection should be followed up for at least 2 years for evidence of neurodevelopmental delay, regardless of the initial presentation. 62

#### *CSF analysis*

CSF pleocytosis may occur in CHIKV encephalitis. 62 As with other viral CNS infections, CSF lactate is below 3.5 mmol/L and glucose under normal. 71 72 Some cases with CHIKV meningitis showed a predominance of polymorphonuclear cells. 73 CSF pleocytosis has been found in 50% of CHIKV myelitis cases. 62 The typical cytoalbuminological dissociation CSF was found in 80% of the GBS cases. 65 Specific intrathecal IgG synthesis was found in a case of CHIKV encephalitis. 74

#### *CHIKV tests*

In neuro-CHIKV serum tested positive for CHIKV infection in all 64 cases, and CSF tested positive in 31/33 cases. 65 Because CHIKV is an alphavirus, there is no serological cross- reactivity with the flaviviruses (DENV and ZIKV), making diagnosis more straightforward (in areas where other alphaviruses are not circulating).

In encephalitis, RT-PCR was positive in 62%, and IgM in 80% (12/15). In GBS, RT-PCR was typically negative and IgM positive of CSF. In ADEM, optic neuropathy, and neuroretinitis, serum IgM was positive and RT-PCR negative, where tested. The virus can also be detected in urine, saliva, semen, and milk but the same caveats apply. 75 76 77 CHIKV RNA can be detected using unbiased metagenomic next-generation sequencing (mNG). 73

#### *Neuroimaging*

MRI in encephalitis was altered in 83% of the cases. CHIKV encephalitis in adults and children does not appear to show a distinct pattern. 62 Many cases had normal imaging. 74 78 79 In spinal cord disease, MRI may range from changes suggestive of demyelinating lesions to extensive T2/fluid-attenuated inversion recovery hyperintensity. 80

### Management

Although various in vitro compounds active against CHIKV have been reported, there are currently no specific antiviral agents (WHO 2022). 81 No antiviral has been evaluated in the management of CHIKV-associated neurological disease, which therefore remains the same as that of neurological disease without associated CHIKV infection. The development of the CHIKV vaccine appears to be the best strategy for controlling outbreaks. 82

In conclusion, in recent years, there has been great progress in the knowledge of arboviruses with potential neuroinvasive and neurological complications, especially in Brazil, where neurologists and researchers from related areas have performed numerous research and publications on the subject. It has long been known that arboviruses represent an important cause of encephalitis. However, twenty years ago, many doctors were unaware of the numerous other manifestations, such as autoimmune diseases that occur in re-emerging infections caused by the DENV (GBS, ADEM, and neuromyelitis optica).

On the other hand, neurological diseases caused by the emerging CHIKV and ZIKV have had a new and significant impact on human health due to their severity and the potential to cause sequelae and death. Both infections were not restricted to affecting adults, but also to fetuses through maternal-infant infection. These viruses have been a cause of congenital malformation syndromes with great social and economical repercussions.

The early diagnosis of these arbovirus infections in the nervous system has been made possible by scientific advances in the areas of immunology and molecular biology, with the detection of specific antibodies and the viral agent in the blood, CSF, in addition to other fluids and tissues. Although to date, there is no specific anti-viral treatment, early symptomatic measures can save lives. The development of vaccines and vector control through biotechnological techniques and population behavior are the most promising ways arboviruses and its neurological complications. There are currently dengue vaccines that are effective against all four serotypes.

Considering that neuroinvasive arboviruses represent a significant burden on public health in endemic areas, government support in the development and distribution of vaccines as well as vector control programs is essential. The impact on the incidence of diseases associated with neuroinvasive arboviruses is expected to increase further due to climate change and urbanization. Such environmental changes may lead to the expansion of the vector and these viruses worldwide.
